# Excessive Self-Medication with Prescription NSAIDs: A Cross-Sectional Study in Kosovo

**DOI:** 10.3390/pharmacy12030093

**Published:** 2024-06-12

**Authors:** Gentiana Krasniqi, Ilirjeta Qeriqi, Genta Qeriqi, Rajmonda Borovci, Daniela Zenelaj, Fehmi Rrahmani, Manushaqe Kryeziu-Rrahmani, Nderim Kryeziu

**Affiliations:** 1 Alma Mater Europaea Campus College “Rezonanca”, 10000 Pristina, Kosovo; 2 Klinikum Lippe Detmold, 32756 Detmold, Nordrhein-Westfalen, Germanymanushaqe.kryeziu@yahoo.de (M.K.-R.)

**Keywords:** adverse reactions, community pharmacy, NSAID, self-medication

## Abstract

Non-steroidal anti-inflammatory drugs (NSAIDs) are commonly used to manage pain, fever, and inflammation. Although most are usually classified as prescription-only medicines, in many countries they are frequently purchased for self-medication purposes. This study explores NSAID-usage patterns in Kosovo, aiming for a safer and more effective medicinal use. The study employed a dual approach to collect data. First, NSAID sales were analyzed in a convenience sample of ten community pharmacies across diverse regions in Kosovo in 2023. Second, data on NSAID-usage patterns and patient awareness were systematically gathered from 410 patients during routine pharmacist–patient interactions. The four most commonly purchased NSAIDs according to sales analysis were diclofenac (33.1%), ketoprofen (27.6%), ibuprofen (17.0%) and nimesulide (12.7%). A significant 74.8% of NSAIDs were bought without prescriptions, particularly among younger adults (20–39 years), who accounted for 82.8% of such purchases. The predominant reason for NSAID use was headache (43.8%). Although many of the patients suffered from occasional (33.7%) or frequent (12.6%) stomachaches and took acid-lowering medicines, the majority (85.9%) could not recall any NSAID adverse reactions. This study exposes widespread self-medication and a significant lack of awareness regarding potential risks of NSAIDs, particularly among young adults. To address these issues, it is critical to improve dispensing practices through increased pharmacist awareness and stricter law enforcement.

## 1. Introduction

Non-steroidalanti-inflammatory drugs (NSAIDs) are widely used to alleviate pain, fever, and inflammation [1,2,3]. However, their use is sometimes limited due to drug interactions and adverse reactions, including gastrointestinal disturbances and cardiovascular, kidney, and liver toxicity [4,5,6]. A systematic review identified NSAIDs among the top 10 drug classes associated with fatal events, and third in terms of non-fatal events [7]. Even at low doses, the prolonged use of NSAIDs can lead to adverse reactions [8]. While most NSAIDs have a comparable safety profile, certain adverse reactions are specific to particular drugs [4,9]. Therefore, selecting an appropriate NSAID tailored to the individual patient’s circumstances is crucial to ensuring both efficacy and safety [1,10].

Despite the global popularity of ibuprofen, diclofenac and naproxen, there are variations in NSAID utilization across different countries [11,12,13], which may not always be based on scientific evidence [14]. For instance, nimesulide is not marketed in the USA, Germany, France, and some other EU countries, but is extensively utilized in Italy, Greece, Serbia, and many other European countries [6,11,12,13,15]. Nimesulide is a good example, as its use is restricted by the EMA as a second-line treatment with an obligation upon the Marketing Authorization Holders to inform health care professionals of the safety risks [16]. According to the Medicines Agency in Kosovo, there were 19 NSAIDs marketed in Kosovo in 2023 (https://akppm.rks-gov.net/; accessed on December 2023), all of which were listed as prescription-only, except for ibuprofen and acetylsalicylic acid, which were available over-the-counter (OTC) [17].

In developing countries like Kosovo, with neither a public health insurance system nor a reimbursement program, patients may tend to self-medicate by purchasing medicines directly from community pharmacies without a prescription. Currently, Kosovo’s legislation (MSH-11/2015-UA) only imposes financial penalties for non-professional behavior and does not include license suspension for pharmacists who sell prescription-only medicines without a prescription. Moreover, as of May 2024, Kosovo had 866 licensed community pharmacies, 137 licensed pharmaceutical wholesalers for medicinal products, and 80 for medical devices (https://akppm.rks-gov.net/; accessed on 25 May 2024). Despite this extensive network, regulatory enforcement faces significant challenges due to an insufficient number of pharmaceutical inspectors. Until recently, there were only 12 inspectors, with 8 more recruited in April 2024, bringing the total to 20 (https://msh.rks-gov.net/; accessed on 25 May 2024). However, this number remains inadequate to effectively oversee such a large network, underscoring the urgent need for enhanced regulatory mechanisms and oversight.

The objectives of this study were as follows:

1. To investigate the utilization pattern of NSAID medications;
2. To assess patient characteristics and their level of awareness regarding NSAID medications.

## 2. Materials and Methods

### 2.1. Study Design and Setting

This cross-sectional study was conducted from 1 January 2023, to 31 December 2023, across ten community pharmacies in Kosovo. These pharmacies were selected through convenience sampling and were located in six of the seven administrative districts of Kosovo, including three in the capital city, Pristina. While ensuring geographical representation through a diverse sample from various regions, the pharmacies were selected based on established professional networks and their willingness to participate. The study aimed to analyze NSAID sales data and collect anonymized patient information during routine pharmacist–patient interactions to assess NSAID use patterns and patient awareness of NSAID medications.

### 2.2. Ethical Considerations

The study was conducted in accordance with the Declaration of Helsinki, and the protocol was approved by the College Ethics Committee (AD-1315-1/23). All subjects gave their informed consent for inclusion before they participated in the study. No personal identifiers were collected, ensuring the confidentiality of participant data.

### 2.3. Data Collection

Sales data collection and management: Each participating pharmacy provided detailed sales records for NSAID-containing products, which were extracted using Pro Data Finance software (http://prodata-ks.com/; accessed on 25 May 2024). Only oral and suppository dosage forms intended for adult use were considered. The total quantity of each specific NSAID sold was aggregated from different manufacturers to determine the number of defined daily doses, which was then used to calculate utilization percentages for each pharmacy individually (https://www.who.int/tools/atc-ddd-toolkit/indicators, accessed on 25 May 2024). These percentages from 10 pharmacies were then averaged to provide a national overview of NSAID utilization. The data are presented as mean values with standard deviations. Additionally, the cumulative daily doses sold for each NSAID are presented, enabling an assessment of the volume of data used for analysis.

Collection of patient data: In November 2023, pharmacists gathered key information from adult customers (aged 20 years and older) purchasing NSAIDs, utilizing a standardized data collection form. Developed under the guidance of the College’s Scientific Committee, this form was refined through a pilot study with 10 patients per pharmacy to optimize data collection. Designed to integrate seamlessly into daily pharmacy operations, the form collected essential details such as the patient’s sex, age, district, type of NSAID purchased, possession of a medical prescription, indications for NSAID use, awareness of adverse reactions, and use of acid-lowering drugs. Pharmacists were trained to efficiently incorporate this form into their interactions with patients. Initially filled out on paper, the forms were then entered daily into an online Google Form to facilitate continuous monitoring and immediate feedback. To minimize data inaccuracies, only adult customers purchasing NSAIDs for themselves were approached. The initial sample size calculation using Raosoft software (Sample Size Calculator; Raosoft Inc., Seattle, WA, USA) estimated that 385 patients would be needed; however, a total of 509 consecutive patients were approached to ensure the robustness of the data. Of these, 73 patients did not consent to participate, resulting in a response rate of 86% (436 patients). After excluding 26 due to incomplete data, 410 patients were included in the final analysis.

### 2.4. Statistical Analysis

Data were analyzed using SigmaPlot 14.0 (Systat Software, Inc., San Jose, CA, USA). For sales data, the statistical significance of the differences between NSAIDs sold in pharmacies was assessed using One-way Repeated Measures ANOVA. For patient data, categorical variables were summarized using frequencies and percentages, and associations were tested using the Chi-square test. A *p*-value of <0.001 was considered statistically significant.

## 3. Results

### 3.1. Overview of Annual NSAID Sales Patterns

**Figure 1:**
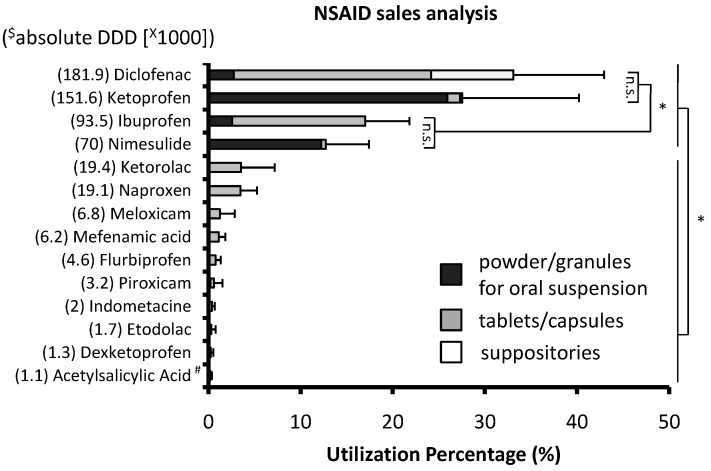
Utilization percentage based on sales of marketed NSAID products: One year sales analysis (2023) from 10 community pharmacies throughout Kosovo was used to estimate relative NSAID utilization. (*n* = 10, mean + SD; One-way RM ANOVA; * *p* < 0.001). ^#^ dispersible tablets; ^$^ absolute DDD: the cumulative number of DDDs sold for each NSAID in all participating pharmacies.

Among 19 different NSAIDs marketed in Kosovo, diclofenac was the most purchased (33.1 ± 9.8 %DDD), followed by ketoprofen (27.6 ± 12.6 %DDD), ibuprofen (17.0 ± 4.8 %DDD) and nimesulide (12.7 ± 4.7 %DDD) (Figure 1). Together, these four drugs accounted for over 90% of NSAID drug utilization (DU90%). Ketoprofen and nimesulide were predominantly purchased in the form of powder/granules for oral suspension (>94%). These four NSAIDs were purchased significantly more than all other NSAIDs (One-way RM ANOVA, *p* < 0.001). At much lower quantities, ketorolac and naproxen ranked fifth and sixth, whereas celecoxib and etoricoxib (selective COX-2 inhibitors) were purchased in negligible amounts (thus not shown in the graph).

### 3.2. Patient Demographics and NSAID Purchasing Behaviours

Out of the total 410 patients, 209 (50.9%) were females and 201 (49.1%) were males, representing various age groups (refer to Table 1). The majority of NSAIDs users (approximately 62%) were younger than 39 years old, reflecting the youthful population of Kosovo. Around 42% of the patients were from the capital city, Pristina, while the remaining participants resided in other districts.

Similar to the one-year sales analysis, diclofenac, ketoprofen, ibuprofen, and nimesulide emerged as the top four NSAIDs, accounting for purchases made by 93% of the patients (Table 1). Therefore, the subsequent detailed analysis of the patient data focuses on these four NSAIDs. Only 25.2% of the patients purchased NSAIDs based on a physician’s prescription (Table 1). The majority either self-medicated by specifically requesting a particular product by name without consulting the pharmacist (64.6%) or sought the pharmacist’s suggestion after explaining their medical condition (10.2%).

**Figure 2:**
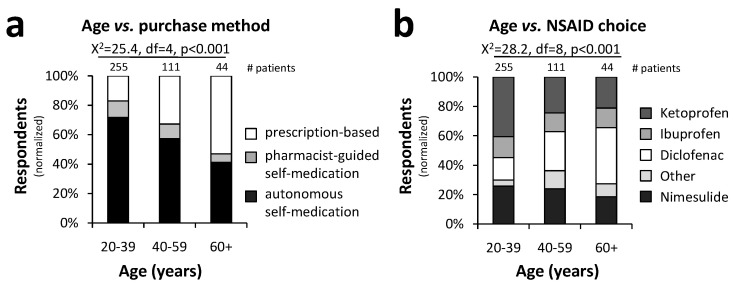
The impact of the patient’s age on NSAID choice and purchase method: Younger patients purchased NSAIDs mostly for self-medication (**a**), preferably nimesulide and ketoprofen (**b**). Self-medication was less prevalent in older adults (**a**), while diclofenac was the main NSAID purchased by patients older than 60 years (**b**) (statistical test: Chi-square test; *p* < 0.001).

Self-medication was found to be more prominent among younger adults. In fact, 82.8% of the patients aged between 20 and 39 years did not visit a physician and required NSAIDs without a medical prescription (Figure 2a). The proportion of self-medication decreased with age, down to 47% for patients over 60 years of age (Figure 2a). Among the younger patients, ketoprofen and nimesulide were the preferred choices (40.6 and 25.8%, respectively) (Figure 2b). In contrast, patients showed an increased preference for diclofenac with age (20–39: 15.3%; 40–59: 26.5%; 60+: 38.0; Figure 2b). The Chi-square test demonstrated a significant association between age groups and purchase method (X^2^ = 25.4, df = 4, *p* < 0.001), as well as age groups and NSAID type (X^2^ = 28.2, df = 8, *p* < 0.001).

### 3.3. Dosage form Preferences by Age and Prescription Status

**Figure 3:**
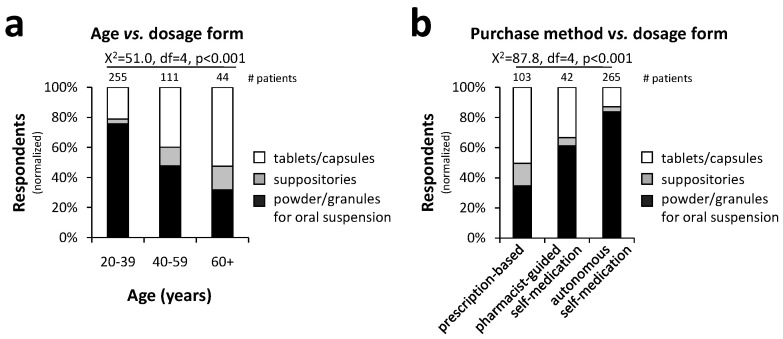
Impact of age and purchase method on NSAID dosage form preferences: Younger patients (20–39 years) predominantly choose powders/granules, whereas older patients (over 60) preferred tablets/capsules; suppositories became more popular with increasing age (**a**). While pharmacists’ recommendations aligned more with patient preferences for powders/granules, doctors prescribed tablets/capsules more often (**b**) (statistical test: Chi-square test; *p* < 0.001).

The choice of pharmaceutical form varied significantly with the patient’s age and method of NSAID purchase, as shown in Figure 3a. Younger patients, aged 20–39, predominantly chose powder/granules for oral suspensions (75.7%) over tablets and capsules (21.1%). In contrast, older patients above 60 years showed a lower preference for powder/granules for oral suspension (31.8%) and a higher preference for tablets/capsules (52.6%). Although suppository dosage forms were generally the least preferred across all age groups, their popularity increased with age, being nearly five times more favored by patients older than 60 (15.6%) than those younger than 39 (3.2%). The Chi-square test demonstrated a significant association between patient age and dosage form (X^2^ = 51.0, df = 4, *p* < 0.001).

There was a noticeable trend in dosage form preferences depending on the recommender—medical doctors, pharmacists, or the patients themselves (Figure 3b). Patients strongly favored powders/granules for oral suspension, with 83.8% selecting this form. Recommendations from pharmacists also leaned towards powders/granules, with 61.1% favoring this form. In contrast, upon examining the doctors’ prescriptions, we found that NSAIDs in the form of powders/granules for oral suspension were prescribed in only 34.7% of the cases. Suppositories were the least popular choice overall; however, they were prescribed by doctors at a rate nearly four times higher (14.9%) than patients’ selections (3.4%). A Chi-square test revealed a significant association between the purchase method and the dosage form selected (X^2^ = 87.7, df = 4, *p* < 0.001).

### 3.4. Indications for NSAID Use and Purchase Patterns

**Figure 4:**
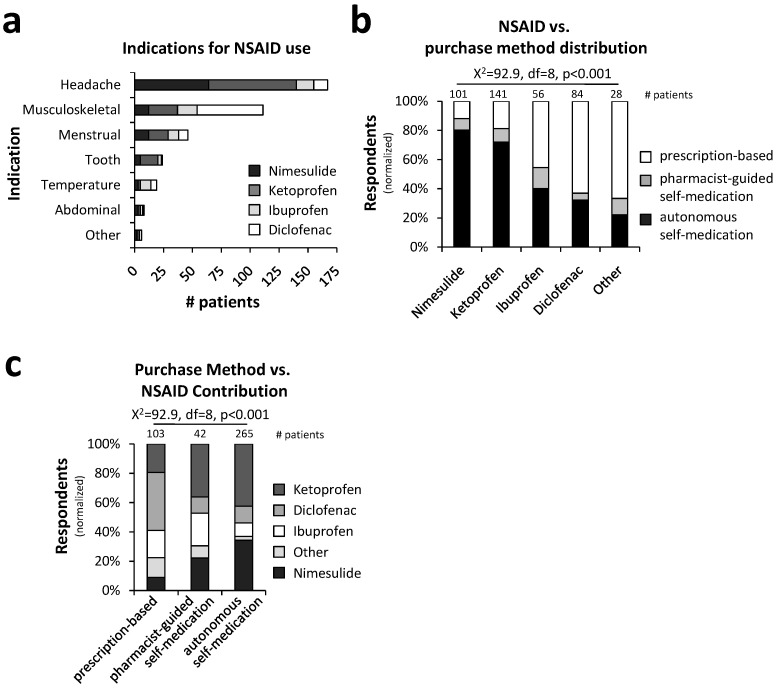
Trends in NSAID indications and purchase methods: Headache was the main indication for NSAID purchase, and it was mainly treated with nimesulide and ketoprofen (**a**). Both drugs were primarily purchased for self-medication (**b**), while physicians mostly prescribed diclofenac and ibuprofen (**c**) (statistical test: Chi-square test; *p* < 0.001).

A considerable portion of the patients (43.8%) reported using NSAIDs primarily to alleviate headaches, as shown in Figure 4a. Within this group, ketoprofen and nimesulide were the most frequently purchased medications, collectively accounting for approximately 84% of the treatments for headaches. The second most common indication for NSAID use was musculoskeletal pain, reported by 29.1% of patients. Menstrual pain also emerged as a significant indication, affecting 12.1% of the sample (Figure 4a).

Overall, the use of nimesulide and ketoprofen was predominantly associated with self-medication, in 88.1% and 81.3% of the cases, respectively (Figure 4b). In contrast, diclofenac was primarily obtained through prescription, accounting for 66.7% of its purchases. Analyzing the same data from the perspective of the method of purchase (Figure 4c) reveals that doctors most frequently prescribe diclofenac (39.6%), whereas patients predominantly self-medicate with ketoprofen (42.4%) and nimesulide (34.3%). Recommendations by pharmacists, while generally aligning more closely with patient preferences, tend to fall between patient choices and medical prescriptions. The Chi-square test demonstrated a significant association (*p* < 0.001) between the tested groups and categories (refer to Figure 4 for further details).

### 3.5. Self-Medication Patterns with NSAIDs by Age and Frequency of Use

**Figure 5:**
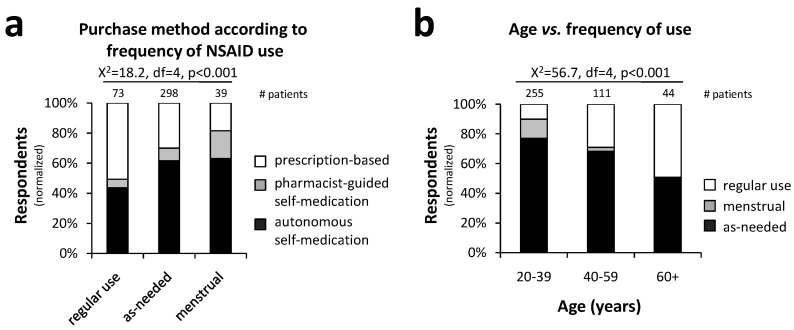
Frequency of NSAID use according to patient age and purchase method: Self-medication was common in all frequencies of use, although less common among regular NSAID users (**a**). Regular NSAID use was more prevalent in the elderly (**b**) (statistical test: Chi-square test; *p* < 0.001).

Figure 5a shows that self-medication was prevalent among patients who purchased NSAIDs for an as-needed use (70.1%) and menstrual pain (81.6%) compared to those who use them regularly (49.3%) (X^2^ = 18.2, df = 4, *p* < 0.001). Additionally, Figure 5b illustrates that regular NSAID use increased with age, from 10.2% in patients aged 20–39 to 49.2% in those older than 60 (X^2^ = 56.7, df = 4, *p* < 0.001).

### 3.6. Patient Awareness and Health Outcomes

**Figure 6:**
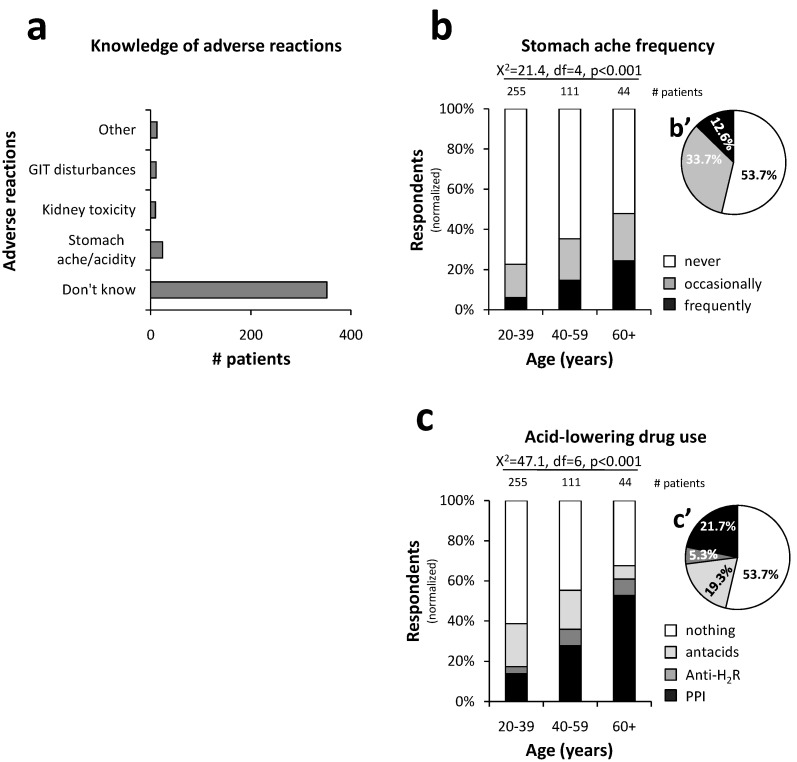
Patient awareness of NSAID adverse reactions and stomach complaints: The majority of the patients were not aware of any adverse reactions to the NSAID they were purchasing (**a**). Stomachache and anti-acid drug use are presented according to age groups (bar graphs (**b**,**c**), respectively), as well as for all age groups together (pie charts (**b′**,**c′**), respectively). Many patients experienced stomachaches (**b**), for which they took acid-lowering drugs (**c**). Both stomachaches and acid-lowering drug use increased with age (**b**,**c**). (PPI: proton pump inhibitor; Anti-H_2_R: Histamine 2 receptor antagonist) (statistical test: Chi-square; *p* < 0.001).

Despite the high prevalence of self-medication, 85.9% of the patients were unable to recall any adverse reactions associated with the NSAID they were purchasing (Figure 6a). Less than 2.5% associated NSAIDs with risks to the kidneys, while only 5.9% of the patients made a direct connection between the NSAID-use and the occurrence of adverse reactions in the stomach. Nevertheless, many patients reported experiencing occasional (33.7%) or regular (12.6%) stomachaches (Figure 6b′), and these complaints increased significantly with the age of the patients (X^2^ = 21.4, df = 4, *p* < 0.001; Figure 6b). Correspondingly, 21.7% of the patients reported taking proton pump inhibitors, another 5.3% H_2_ anti-histaminic drugs, and 19.3% antacids (Figure 6c′) at least once a week. The use of acid-lowering drugs also increased significantly with age (X^2^ = 47.1, df = 6, *p* < 0.001; Figure 6c).

## 4. Discussion

### 4.1. NSAID-Usage Patterns in Kosovo

Our study identifies diclofenac, ketoprofen, ibuprofen, and nimesulide as the most commonly used NSAIDs in Kosovo, based on both sales data and patient reporting.

Compared to global trends, we observe similarities with countries like Italy, where diclofenac and nimesulide lead (17.9 vs. 16 %DDD, respectively), and ketoprofen follows ibuprofen (10.6 vs. 14.4 %DDD, respectively) [11,18,19]. However, this contrasts with wider EU patterns where diclofenac and ibuprofen are universally popular, while nimesulide and ketoprofen usage varies by country. Nimesulide is not marketed in Germany, and due to hepatotoxicity concerns, its use has been restricted by the EMA [16] and therefore withdrawn from the market in Spain, Finland, Belgium, France, and Ireland [4,16,20,21]. Similarly, ketoprofen is seldom used in Germany, the UK, the Netherlands, and Denmark [11,18,19]. A different picture arises in the USA, where ibuprofen and naproxen are the preferred NSAIDs [20]. Such diverse national preferences are evident even within smaller regions like the Balkans: nimesulide ranked third in Serbia but saw limited use in Croatia, where ketoprofen was more common [18,22].

These discrepancies suggest that NSAID selection is influenced by local factors beyond medical evidence and cost-effectiveness, as demonstrated by a study in Croatia [14]. In Kosovo, the high level of self-medication and low awareness of adverse reactions also make it unrealistic to expect that NSAID selection is based on evidence, as people often choose medications on their own rather than relying on medical advice. Contrary to the conclusion from the study in Croatia [14], the prominent purchase of single-dose sachets with powder/granules for oral suspension may actually be perceived as a cheaper alternative. While a sachet typically costs more than a tablet, purchasing a few sachets for temporary use is more economical than buying a 30-tablet box. Finally, despite a logical expectation that over-the-counter medications like ibuprofen and acetylsalicylic acid would dominate in a setting with such a high proportion of self-medication, it is actually prescription NSAIDs such as diclofenac, nimesulide, and ketoprofen that are most frequently used.

### 4.2. Patterns and Implications of NSAID Self-Medication in Kosovo

Despite the heterogeneity in preferred NSAIDs between countries, self-medication with various types of medicines remains a common global issue, extending beyond underdeveloped and developing nations [15,23]. For example, a study in Greece, an EU member state, found that 44.6% of surveyed patients in rural areas obtained antibiotics without a prescription [24]. According to a study on antibiotic abuse in Kosovo, cost minimization and time saving were the primary reasons for avoiding medical consultation [25]. The absence of a public health insurance and drug reimbursement system in Kosovo provides little financial incentive for patients to seek prescriptions, encouraging self-medication practices.

Our study also shows that a significant portion of NSAID users are younger individuals, reflecting the country’s youthful demographic profile, with less than 10% of the population over 65 years of age [26]. We show that this younger demographic commonly uses NSAIDs for self-medicating conditions such as headaches, musculoskeletal pain, and menstrual pain. Their preference for immediate relief is particularly evident in their choice of NSAIDs like ketoprofen and nimesulide in powder or granule forms, which are potentially perceived as acting faster and stronger. This trend indicates a prioritization of quick pain relief over potential long-term health risks.

The trend of self-medication is particularly concerning due to the associated low awareness of potential adverse reactions among NSAID users. The overwhelming majority of patients are not only unaware of these risks but also fail to associate their gastrointestinal symptoms with the NSAID usage [16,27]. This significant knowledge gap underscores the critical need for public health initiatives aimed at educating patients about the safe use of medicines and the importance of professional medical advice.

### 4.3. The Role of Community Pharmacies in Mitigating Self-Medication and Enhancing Drug Safety

Community pharmacies play an indispensable role as the last point of contact and primary healthcare providers for patients. Pharmacists are in a unique position to consult with patients during medication dispensing, providing a crucial opportunity to understand their needs and enhance awareness through education. While recognizing the issue of self-medication, many pharmacists perceive it as a firmly established societal norm, which discourages them from taking proactive measures [25]. Moreover, the commercial nature of community pharmacies may exacerbate the issue of self-medication. Pharmacists may often yield to patient demands to avoid losing business to competitors, often located just a few meters away, rationalizing that individual actions are unlikely to make a significant impact on a culturally entrenched practice [25].

Our findings reveal an interesting positioning of the pharmacists, particularly in dispensing ketoprofen and nimesulide. While these drugs are commonly chosen for self-medication and seldom prescribed by physicians, pharmacists seem to promote a more balanced approach. This tendency likely indicates their efforts to reconcile patient preferences with appropriate medical oversight. Such actions emphasize the critical role of pharmacists in bridging the gap between professional medical advice and patient choices, ultimately enhancing drug safety and improving NSAID management.

However, the prevalent self-medication with prescription-only medicines implicates pharmacists in problematic professional and legal behavior. Therefore, it is essential to enhance pharmacists’ awareness and motivation to adhere to good dispensing practices through structured initiatives, professional education programs, and appropriate financial incentives. Furthermore, regulatory authorities must not only enforce existing laws more rigorously, but also consider strengthening the mechanisms of law enforcement on drug dispensing. Currently, legislation in Kosovo (MSH-11/2015-UA) primarily imposes only financial penalties for non-compliance, which may not be sufficient to deter malpractices effectively. Our findings underscore the need for more stringent enforcement measures, including potentially revoking the working licenses of violators, to ensure professional discipline and uphold safety standards.

### 4.4. Study Limitations

The data for this study were derived from sales analyses of community pharmacies and NSAID utilization from patients. While geographical diversity was attempted by including pharmacies from six out of the seven administrative districts of Kosovo, and potential seasonal variations were mitigated through sales data spanning a one-year period, our study has several limitations. First, the sample included only ten community pharmacies, chosen through convenience sampling due to the willingness of pharmacies to reveal business volume and disclose practices involving the sale of prescription-only NSAIDs without prescriptions. This selection method may potentially skew NSAID-usage patterns and limit the generalizability of our findings. Moreover, the informal method of data collection during routine pharmacist–patient interactions might also introduce variability in the accuracy and depth of the data collected. And finally, the study’s design did not incorporate the perspectives of pharmacists. The sample of the pharmacists involved in dispensing NSAIDs in the 10 participating community pharmacies was very small to draw reliable conclusions. The omission of this viewpoint might overlook key factors influencing NSAID consumption patterns and compliance with prescription regulations, and should be further addressed in future studies.

Our methodology was compelled to rely on data from community pharmacies due to the unavailability of comprehensive national data. Despite our efforts to obtain detailed records on drug imports, usage, and pharmacovigilance reports from the Medicines Agency of Kosovo, these requests were not fulfilled. The absence of such critical information not only constrained the scope of our study but also presents a significant challenge in monitoring and regulating pharmaceutical practices effectively.

## 5. Conclusions

This study highlights the urgent need to improve practices and awareness regarding NSAID use in Kosovo, with implications that may extend to other developing and underdeveloped countries globally. We have identified the most commonly used NSAIDs and revealed concerning trends of self-medication and a limited awareness of potential adverse reactions, especially among younger adults. Contributing factors to these issues include inadequate patient awareness, a lack of financial incentives for patients to obtain prescriptions, a profit-driven focus within community pharmacies, and lenient law enforcement. By implementing stricter professional standards, improving patient education, and increasing the involvement of pharmacists, we can promote a safer and more accountable healthcare environment. Additionally, it is essential for governmental and regulatory agencies to enhance their data sharing practices. Such improvements are vital to ensuring effective oversight and addressing the challenges facing the health system.

## Figures and Tables

**Table 1:** General characteristics of the NSAID-using patient sample.

Patients’ Characteristics	# Patients (%)
**Age (years**)	
20–39	255 (62.2)
40–59	111 (27.2)
60+	44 (10.6)
**Sex**	
Male	201 (49.1)
Female	209 (50.9)
**Districts**	
Pristina (capital)	173 (42.2)
Other districts	237 (57.8)
**Medicine purchased**	
Ketoprofen	141 (34.4)
Diclofenac	84 (20.5)
Nimesulide	101 (24.6)
Ibuprofen	56 (13.7)
Other	28 (6.8)
**Purchased method**	
Prescription-based	103 (25.2)
Pharmacist-guided self-medication	42 (10.2)
Autonomous self-medication	265 (64.6)

## Data Availability

The datasets generated and/or analyzed during this study are not publicly available due to the use of anonymous data (community pharmacies and patients); however, datasets are available from the corresponding author on reasonable request.
